# Beneficial Effects of Physical Activity on Subjects with Neurodegenerative Disease

**DOI:** 10.3390/jfmk5040094

**Published:** 2020-12-16

**Authors:** Laura Vizzi, Elvira Padua, Agata Grazia D’Amico, Virginia Tancredi, Giovanna D’Arcangelo, Ida Cariati, Manuel Scimeca, Grazia Maugeri, Velia D’Agata, Michela Montorsi

**Affiliations:** 1 Department of Human Sciences and Promotion of the Quality of Life, San Raffaele Roma Open University, 00166 Rome, Italy; laura1vizzi@gmail.com (L.V.); elvira.padua@uniroma5.it (E.P.); michela.montorsi@uniroma5.it (M.M.); 2 Department of Drug Science, University of Catania, 95125 Catania, Italy; agata.damico@unict.it; 3 Department of Systems Medicine, “Tor Vergata” University of Rome, 00133 Rome, Italy; giovanna.darcangelo@uniroma2.it; 4 Centre of Space Biomedicine, “Tor Vergata” University of Rome, 00133 Rome, Italy; 5 Department of Clinical Sciences and Translational Medicine, “Tor Vergata” University of Rome, 00133 Rome, Italy; ida.cariati@uniroma2.it; 6 Department of Biomedicine and Prevention, “Tor Vergata” University of Rome, 00133 Rome, Italy; manuel.scimeca@uniroma2.it; 7 Sections of Human Anatomy and Histology, Department of Biomedical and Biotechnological Sciences, University of Catania, 95123 Catania, Italy; grazia.maugeri@unict.it (G.M.); vdagata@unict.it (V.D.)

**Keywords:** physical activity, dementia, neurodegenerative process

## Abstract

Studies on the effectiveness of physical exercise to treat and/or prevent mental disorders are essential and particularly appropriate, given the rapid growth of the elderly population and the consequent increase in the prevalence of neurodegenerative diseases. The onset of neurodegenerative diseases is subtle, and progression is irreversible, as there is still no cure capable of stopping them permanently. Therefore, we should not underestimate these diseases and should immediately begin to combine the treatment with physical activity adapted to specific needs. Indeed, it is well known that physical activity has positive effects on mobility, autonomy, and functional capacity, improving not only cognitive functions, but also reducing the risk of developing dementia. Despite several studies in this field, to date there are no specific and effective protocols that promote physical exercise in people with dementia. Based on this evidence, the aim of the present work was to verify whether an adapted physical exercise regimen could promote the maintenance of psychomotor functions in elderly subjects and, therefore, delay the irreversible effects of combinations of dementia and other pathologies associated with aging. Our results clearly show that exercise is very effective in improving psychomotor functions and delaying the progress of neurodegenerative diseases in humans, since we observed that the subjects maintained their cognitive skills after 8 months of physical activity, moreover, two patients presented an amelioration. Based on the results obtained, we recommend that the motor practice, in any chosen form, be considered an integral part of prevention programs based on an active lifestyle in older people. Future studies will be necessary to establish how long lasting the benefits of a specific physical activity are and whether they are enough to delay cognitive decline.

## 1. Introduction

Aging and cognitive decline are two of the main causes that lead to the onset of neurodegenerative disease and are identified through a progressive evolution of brain and motor symptoms. Aging occurs at different rates across subjects. Therefore, it is difficult to establish a generalized pattern [1]. Aging also depends on the damage linked to the coexistence of different diseases or lifestyles due to physiological aging [2]. Physiological aging cannot be defined or measured, since it can be influenced by different genetic factors. Nevertheless, risk factors such as smoking, excessive alcohol consumption, and unhealthy lifestyles can increase the onset of cognitive decline [3].

The World Health Organization (WHO) defines aging or senescence as a period of life in which the loss of mental and/or physical functions becomes more manifest and established the referred age at 65 years old [4]. The prevalence and incidence estimate of cognitive decline vary across studies [5] in relation to the diagnostic criteria used for defining cognitive decline, the sample population, and the diagnostic procedures. The elderly typically suffers from concomitant multiple diseases that expose them to a risk of loss of autonomy (body size and composition, cardiovascular, respiratory, and brain functions). It has been demonstrated [5] that all the capacities of our body decrease with aging in different ways [6].

Physical activity and exercise can improve mobility, autonomy, and functional skills. Moreover, cognitive functions have positive effects on the quality of life and well-being [7,8]. Physical activity improves health and reduces the incidence risk of coronary heart disease, stroke, some kinds of cancer, type 2 diabetes, obesity, hypertension, osteoporosis, falls, and mortality [9]. Exercise can be effective in improving cognition, independent functioning, and psychological health in adults over 65 and sedentary adults with the diseases mentioned above [10,11]. A recent review [12] highlighted the beneficial effects of exercise on cognition and brain health in the elderly, suggesting a possible correlation between the improvement of cardiorespiratory activity and cognitive functions. According to the United States Centers for Disease Control and Prevention [13], 150 min/week of moderate-intensity aerobic exercise are enough to reduce the probability of acquiring the vascular risk factors mentioned above.

The aim of this research was to determine if the adapted physical exercise can promote the maintenance of the psychomotor functions in elderly subjects, or delay the irreversible effects linked to dementia and other pathologies associated with aging. The main aging-associated diseases evaluated in this study were obesity, oxidative stress (the production of free radicals), dementia, high blood pressure, diabetes mellitus, osteoporosis, and sarcopenia.

In this work, we analyzed the effects of 8 months of physical activity on subjects with different kinds of neurodegenerative diseases (defined complex patients).

The Agency for Healthcare Research and Quality defines a complex patient (MCC, Multiple Chronic Conditions) as a subject with two or more chronic diseases, where each of these medical conditions can affect the outcome of treatment of other comorbidities [14]. For this reason, in our study, we used a valuation and specific approach to research models and needs deriving from multiple pathologies.

## 2. Materials and Methods

### 2.1. Participants and Setting

Twenty-six subjects were tested for both evaluative factors and an exercise protocol. Subjects included female (*n* = 13) and male (*n* = 13), aged between 66–92 years. All subjects were affected by one or more neurodegenerative disease, and they are considered complex patients, including Vascular, Dementia, Parkinson’s Disease (PD), Alzheimer’s Disease (AD), Progressive Supranuclear Palsy (PSP), Alcohol-related dementia (ARD), Frontotemporal Neurocognitive Disorder (FTD), and Lewy Body Dementia (LBD). For a better characterization of the participants qualified for the study, we have provided further details on their clinical conditions in Table S1.

The protocol was administered by the same operator for 8 months. The frequency which subjects carried out this activity was provided by the WHO guidelines. All subjects performed activities three times *per* week, with specific and specialized sessions associated with group recreational motor courses. Table 1 shows the identification number, age, and sex for each subject with the related pathological status.

This study received the *consensus* by the Institutional Research Board of the University San Raffaele of Rome, Italy and all subjects gave written informed consent in respecting the ethical principles of the Declaration of Helsinki.

### 2.2. Tests

To determine the initial and final condition of the subjects, we used two validated tests: Noppain Scale and Modified National Institutes of Health Stroke Scale (mNIHSS) [15,16].

The Noppain Scale has been used to evaluate pathology-related pain intensity during an initial, intermediate, and final phase. The scale is divided into three sections with the central part of the scale being the most relevant to the current study, where the field of interest is pain-associated behavior. The datasheet is completed by qualified staff who observed the patients’ behaviors for at least 5 min.

We assigned a score to the pain behavior, as reported in Table 2 (for example, pressure on the pain zone, complaints, restlessness, facial gesturing, rigidity, and support of a body part).

The test is divided into two parts to which a numerical quantification is attributed: In the first part, we reported the total number of “YES” answers resulting from the test; in the second one, we reported the total number of individual intensities. The counted “YES” answers can range from a minimum value of 0 to a maximum value of 6. The intensity score provides a 5-point Likert increasing scale, where 0 corresponds to the lowest possible intensity and 5 to the highest possible intensity, for each action of the test. Therefore, the maximum total value for the intensity score is 30.

Subsequently, we added the values from the first and second part and included everything in the evaluation of the results, which was then associated with the second test of our study.

The mNIHSS is a tool for objectively quantifying the impairment caused by the stroke. Previously, the National Institutes of Health Stroke Scale (NIHSS) [16] was used for this purpose but it was composed of an additional five elements (level of consciousness, best visual, ataxia, dysarthria, and best language), which made the evaluative result unreliable. As shown in Table 3, the mNIHSS consists of 11 objects: Orientation, understanding, and execution of simple orders, gaze, visual field, facial paralysis, left upper limb motility, right upper limb motility, left lower limb motility, right lower limb motility, sensitivity, language, and distraction. Each of these rates a specific ability from 0 to 4. For each object, a score of 0 corresponds to the normal function of the skill, while a higher score is indicative of a certain level of impairment. Finally, the scores for each element are added up to calculate a patient’s total mNIHSS score. The score ranges from 0 to a maximum of 31.

Considering the neuromotor skills, we verified whether any brain damage could irreversibly influence motor skills and abilities, such as loss of vision/orientation in space, extra eye movement, muscle strength, stiffness, and sensory loss.

The clinical aspects most often detected by these tests are: Unilateral motor deficit of face/limbs (arms or legs), homonymous hemianopia, loss of half the visual field, cerebral dysfunction of upper functions (for example, neglect and aphasia) and sensory deficit, hemiparesis, involuntary movements or dystonia (severe or mild).

### 2.3. Adapted Physical Activity Delivery Protocol

The experimental study was conducted at the “San Gabriele Day Center”, Tuglie (LE). We could consult the medical history and pharmacological recruitment schedules, which are often causes of statistical variations in the assessment. Since anamnesis differs among subjects, each general evaluation phase was followed by a specific activity, carried out three days a week. All subjects examined were first visited privately or by an ASL team member, who diagnosed the actual chronic-degenerative dementia. In addition, the subjects were visited weekly by the center’s neurologist.

### 2.4. Adapted Physical Activity (AFA) Protocol Structure

The AFA protocol was structured as follows: 50′ of orientation techniques, 30′ of cognitive exercises, 30′ of training to improve functional skills, activities of daily life (ADL), 30′ of break, 50′ of psycho-motor exercises to stimulate balance, oculomotor coordination, and rhythm. Cognitive exercises are designed to stimulate attention, memory, language, visual-spatial abilities, calculation, and executive control functions.

The motor session began after a specific cognitive activity, which was diversified daily. In some cases, a warm-up such as walking, nordic walking, or cyclette was proposed, with the aim to bring the subject into diaphragmatic breathing.

The warm-up was adapted for subjects which are wheelchair-bound. For example, an assisted exercise bike could be used in which the subject was seated on a chair, to recruit the mobility of the coxo-femoral joint, and perform the exercise in complete safety. The following specific phase of the work was closely related to the pathology.

The term “physical exercise” means guided exercises acting on the joints, muscle tone, balance, and coordination. Physical activity refers to those exercises that involve both motor and cognitive aspects, such as gardening, home activities, hooping, dice toss, bowling, or mini-golf matches. These terms are used by the National Institute of Aging [17].

The specific physical exercises proposed to users concern the mobilization of the head and the cervical spine in lateral inclination and flexion, the mobilization and toning of the upper and lower limbs, the mobility of the wrists and ankles, and exercises for the hands. A summary program of physical exercises and physical activities carried out during the training protocol is shown in Box 1.

If the anamnesis allows exercises of slightly higher difficulty to be carried out, variations in the orthostatic position can be included, such as possible combinations of the following: March on the spot with an alternating movement of legs and arms; lifting of a knee with the opposite arm; flexion of the hip touching the inside of the foot with the opposite hand; walk along a line and/or change direction according to the different color of the line (left—green, right—blue, straight—red); walk with variations in pace (back, forward, lateral); standing (in front of the wall 20 cm away, hands resting on it, abducting the arms on the sagittal plane and keeping them firmly against the wall during the movement); throw the ball and catching it on the fly; pass or throw small objects between two people.

Before the exercise, cognitive activities were carried out: Individualized cognitive stimulation; stimulation of executive functions; recognition of colors, flavors, moods; stimulation of calculation and language functions; Reality Orientation Therapy (ROT), Montessorian Laboratories; Manual laboratories for the maintenance of praxis; sequential exercises (alphanumeric series, days of the week, and months of the year).

Box 1 **Summary program of physical exercises and physical activities carried out during the treatment days.**

Mobilization of the head and cervical spine in lateral inclination, flexion, and rotation: Head rotation (right and left); lower your head until it touches the chest (or whatever is comfortable for the patient); use your hands to move the head first to the right, return to the starting position and then to the left.

Upper limb mobilization: Anterior and lateral elevation of the arms, abduction and adduction on the horizontal plane, internal and external rotation and circular motions.

Toning upper limbs: While sitting with a stick held with both hands, bring it to shoulder height and then stretch your arms forward.

While sitting in a circle, perform exercises for the mobility of wrists and ankles: Rotate the wrist (first the right and then the left) clockwise and counterclockwise; perform the same exercise with both wrists and then with ankles. Exercises for the hands: Hold a sponge ball in your hands and then make the “plier” with your fingers.

Lower limb mobilization: Active extension of the knee; bilateral dorsal and plantar flexion of the foot; mobilization of the flexing spine (while sitting try to touch your feet). Starting with the arms outstretched, bring them first to the right and then to the left (release of the tracks with a stick).

Toning lower limbs: While sitting alternately lift the bent leg (triple flexion reinforcement); lift your foot until you spread out your leg (femoral quadriceps reinforcement).

A path in which three pins are placed in front of each other at a distance. Patients will be given a stick and asked to walk, rolling a ball with the help of the stick between the pins.

Pass the ball to a mate after saying a word that begins with a certain letter, and then say a word that belongs to the same category.

#### Exercise Criteria

1. The proposed work must NOT require a higher energy expenditure, then the guidelines and the training pace must be adapted to the assessed skills of the subject.
2. Exercises must always be carried out with dual “safety guarantees”: Immediate safety to prevent accidents or inconveniences during the lesson, and secondary safety to avoid psychological consequences, emerging after the lesson.
3. Exercises should always be clearly explained, such that the meaning and purpose of the movements are understood by the subject.
4. Exercise regimens must be engaging in order to stimulate an active and participatory motor response.

## 3. Results

The evaluations were always carried out by the same operator. Table 4 shows the results obtained from the two tests administered (Noppain Scale and NIHSS), with individual evaluations associated with each subject.

The same workflow was implemented for both tests: Initial evaluation (START), final evaluation (END), data analysis, drafting of explanatory tables, and considerations. Subject P02 presented a decrease in repetitive will to the change of position and fewer words of pain. Subject P05 showed a decrease in non-contextualized words of pain and the involuntary movement of the left elbow. Finally, subject P24 showed a reduced state of restlessness, thus resetting his final score for this test.

In addition, a minimal but significant improvement in both subject P05 and P11 were reported. Specifically, we observed that P05 improved the understanding and execution of simple orders, while P11 improved the orientation in space with a consequent motility of the lower right limb.

Overall, based on the total values reported in Table 4, only some subjects showed improvements and most of them showed unchanged neuromotor functions.

All measurements were taken at the beginning and at the end of the protocol after 8 months.

## 4. Discussion

The present study aims to investigate the efficacy of the adapted physical activity associated with cognitive training cycles on elderly patients affected by different types of neurodegenerative diseases.

Several studies have shown that exercise increases the activation of upper parietal regions involved in attention and the part of the medial frontal gyrus associated with cognitive control. The studies have also evaluated outcomes in specific areas of functioning [18,19]. These domains are often distinct but related [20], and there is evidence that physical activity can simultaneously support several domains of functioning, including cognition, independence in daily activities, and psychological health/quality of life [21].

It is established that new neurons can be generated in the adult brain through processes known as neurogenesis, which occurs especially in the hippocampus, reducing the effects of neurodegeneration [22]. A positive correlation has been demonstrated between neurogenesis and exercise indicating an increase in the size of the hippocampus in human adults [23]. It has been shown that physical activity in subjects with MCI and dementia can lead to physical and cerebral improvements [24,25].

A recent hippocampal plasticity study assessed long-term cognitive gains after prescribing resistance exercise in patients affected by MCI [26]. The authors showed for the first time that six months of high-intensity endurance exercise not only promotes cognition in people suffering from MCI, but also protects the hippocampus subfields vulnerable to AD from degeneration for at least 12 months after the administration of the exercise protocol.

Resistance training in elderly subjects has been shown to improve muscle strength, balance, and gait after 8 weeks of long-term moderate exercise and reduced the incidence of falls in frail elderly patients with dementia [27]. Therefore, the best strategy to change the morphology and performance of the muscular system is physical exercise [28]. The literature reports evidence about the influence of different aspects of lifestyle on cognitive decline and the risk of dementia [29].

Dementia is a key challenge for future modern society and future research should establish how long-lasting the benefits of specific physical activity are and whether they are adequate to delay cognitive decline. Non-pharmacological and assistance new therapeutic approaches have been found to be of pivotal importance when used in association with drug therapy inducing a significant reduction of the progression of disease, as well as the mitigation of the drug-related side effects. Our results clearly showed that physical activity such as aerobic, strength, or dexterity exercises are very effective in improving the psychomotor functions and in delaying the course of neurodegenerative diseases in humans.

In the preparatory phase, the feasibility of the exercise protocol was verified so that each subject had a margin of adaptation, and therefore, a maintenance of motor functions.

However, it is essential to carry out specific exercises, with a focus on a particular level of attention and concentration. These exercises should also be functional for the performance of normal daily activities. This effect can be obtained with balance exercises and coordination of limb movement, which can be performed by either a free body (especially at the beginning to facilitate people in trouble) or by employing small tools that increase the difficulty and variability through several combinations.

The administration of cognitive activities before the exercise, such as individualized cognitive stimulation, stimulation of executive functions, recognition of colors, flavors, moods, stimulation of calculation, and language functions, was of fundamental importance.

The results of this experimental study showed that exercise was the keystone for maintaining initial motor functions. During the months in which the exercise program was carried out, the study subjects showed no further deterioration of their pathological conditions.

The perception of pain is not always present, but sometimes is also altered. In some cases, the verbal manifestation of pain can be associated with an awareness of the pain itself, leading the elderly person to experience feelings of self-doubt or inadequacy.

In our investigation, we have performed two different tests, Noppain Scale Test and mNIHSS. These tests are not complementary, and they are usually used separately. However, in this study, we have decided to use both to better decodify the responses of our complex patients.

Among the 26 subjects involved in the study, only four were not cooperative (P01 both for Noppain Scale and for mNIHSS; P07, P09, P12 for mNIHSS) due to disabling pathologies associated with dementia, which compromised the reliability of the test. After 8 months, the other patients maintained an unchanged cognitive picture except for two subjects, which showed an improvement in the cognitive function. These results likely reflect an increase in the neuronal representation in different areas of the body due to the plasticity of brain cells.

Since the highly heterogeneity of the studied experimental group, the objective of this work was also to provide suggestions for physical exercises that could be carried out at home under supervision. The positive effects of these exercises manifest in the management of everyday activities, both at home and outside the home where environmental stimuli are greater.

Based on evidence from our study and those reported in the literature, we recommend that the motor practice be considered an integral part of prevention programs based on the active lifestyle in older people [30].

To improve the effectiveness, a healthy lifestyle should include a program of exercise done independently at home. According to our observations, the exercises in the AFA protocol for the treatment of dementia-dependent hypomobility syndromes are useful but not exhaustive. Therefore, it is necessary to integrate these exercises with others aimed at the recruitment of cognitive activities that stimulate the mental function of the people who practice them.

Moreover, it is very important to develop a personalized exercise training program based on the specific individual neurodegenerative diseases and the actual possibility of performing exercises efficiently by the subjects. In this regard, there is a consolidated scientific evidence in agreement that many morbidity, disability, and premature mortality conditions can be prevented through healthy behavior and lifestyles, where physical activity is recognized as a determining factor. If this is true for the healthy person, it is even more true for the ill person, since in many chronic diseases the disabling process is aggravated by a sedentary condition which, in turn, causes functional limitations and further disability. Therefore, the performance of adequate and specific regular physical activity protocols, in combination with cognitive activities, could be the solution to allow beneficial effects on subjects with neurodegenerative diseases.

## Figures and Tables

**Table 1:** Participant characteristics.

Participant	Gender	Age	AD	PD	Vascular	Dementia
P01	F	83				X
P02	M	66	X			
P03	M	72		PSP	X	
P04	F	83	X			
P05	F	84	X			
P06	M	69				X
P07	M	89			X	
P08	F	84			X	
P09	M	82	X			
P10	F	92				X
P11	F	72	X			
P12	F	72	X		X	
P13	M	78		Parkinsonism		X
P14	M	72				FTD
P15	F	75	X			
P16	F	85			X	
P17	F	78	X	Parkinsonism		
P18	M	88			X	
P19	M	79				X
P20	F	82			X	X
P21	M	87		Parkinsonism	X	X
P22	M	71				ARD
P23	F	80		Parkinsonism		X
P24	F	90				X
P25	M	79			X	
P26	M	84		Parkinsonism		X

F: female; M: male; AD: Alzheimer’s Disease; PD: Parkinson’s Disease; PSP: Progressive Supranuclear Palsy; FTD: Frontotemporal Neurodegenerative Disorder; ARD: Alcohol-related dementia; X: membership class.

**Table 2:** Noppain scale test.

Pain Behavior: What Did You See and Hear during Care?							
Pain words	Yes	No	Intensity				
“That hurst”—“Ouch!”—“Stop that!”			1	2	3	4	5
Pain faces	Yes	No	Intensity				
Grimaces—Winces—Furrowed brow			1	2	3	4	5
Pain noises	Yes	No	Intensity				
Groans/Moans—Grunts/Cries—Gasps/Sighs			1	2	3	4	5
Bracing	Yes	No	Intensity				
Rigidity—Protect yourself—Holding			1	2	3	4	5
Rubbing	Yes	No	Intensity				
Massaging effected area			1	2	3	4	5
Restlessness	Yes	No	Intensity				
Frequent Shifting—Rocking—Inability to stay sit			1	2	3	4	5
Scoring	TOT (*)		TOT (**)				

(*) Add up the number of “YES” boxes you checked. (**) Add up the number you circled on the intensity scale. TOT: Total.

**Table 3:** Modified national institutes of health stroke scale (mNIHSS) test.

Item Number	Item Name	Scoring Guide	Patient Score
1b	Level of consciousness (LOC) Questions	Answers both correctly	0
		Answers one correctly	1
		Incorrect	2
1c	Level of consciousness (LOC) Commands	Performs both tasks correctly	0
		Performs one task correctly	1
		Performs neither task	2
2	Gaze	Normal	0
		Partial gaze palsy	1
		Total gaze palsy	2
3	Visual fields	No visual loss	0
		Partial hemianopsia	1
		Complete hemianopsia	2
		Bilateral hemianopsia	3
5a	Left arm	No drift	0
		Drift before 10 s	1
		Falls before 10 s	2
		No effort against gravity	3
		No movement	4
5b	Right arm	No drift	0
		Drift before 10 s	1
		Falls before 10 s	2
		No effort against gravity	3
		No movement	4
6a	Left leg	No drift	0
		Drift before 5 s	1
		Falls before 5 s	2
		No effort against gravity	3
		No movement	4
6b	Right leg	No drift	0
		Drift before 5 s	1
		Falls before 5 s	2
		No effort against gravity	3
		No movement	4
8	Sensory	Normal	0
		Abnormal	1
9	Language	Normal	0
		Mild aphasia	1
		Severe aphasia	2
		Mute or global aphasia	3
11	Neglect	Normal	0
		Mild	1
		Severe	2
TOT			

TOT: Total.

**Table 4:** Summary of all data.

Participants	Noppain Scale		NIHSS	
	Start	End	Start	End
P01	/	/	/	/
P02	16	14	11	11
P03	16	16	24	24
P04	3	3	1	1
P05	20	18	6	5
P06	17	17	21	21
P07	24	24	/	/
P08	4	4	3	3
P09	6	6	/	/
P10	3	3	3	3
P11	4	4	4	3
P12	15	15	/	/
P13	6	6	6	6
P14	5	5	3	3
P15	4	4	2	2
P16	0	0	2	2
P17	3	3	6	6
P18	3	3	3	3
P19	5	5	4	4
P20	7	7	4	4
P21	6	6	10	10
P22	4	4	2	2
P23	2	2	8	8
P24	2	0	1	1
P25	3	2	4	4
P26	8	8	11	11

(/) (not classified).

## Supplementary Materials

The following are available online at https://www.mdpi.com/2411-5142/5/4/94/s1, Table S1: Characterization of the participants qualified for the study.
