# Supplementary material for: Beneficial Effects of Physical Activity on Subjects with Neurodegenerative Disease
Source: J Funct Morphol Kinesiol. 2020 Dec 16;5(4):94. doi: 10.3390/jfmk5040094 (PMC7804865; doi:10.3390/jfmk5040094)
Supplement: Supplementary file 1 [file jfmk-05-00094-s001.pdf]

Table S1. Characterization of the participants qualified for the study.

Participants	Age	Related pathology	Diagnosis
P1	83	Arterial hypertension, hypercholesterolemia, low vision (left eye), osteoporosis, depressive syndrome.	Slowed walking due to problems in the right malleolus. False memories and ideational exchanges that cause aggressive behaviour.
P2	66	Secondary epilepsy, low vision (right eye), depressive syndrome, glaucoma (left eye), hypertension.	Sometimes he presents delusional ideas with consequent behavioural disturbances.
P3	72	Diabetes mellitus and psychomotor slowdown, subdural hematoma regressed, prostatic hypertrophy, hypercholesterolemia.	Walking only with assistance such as feeding and toilet. Problems with swallowing, dysphagia.
P4	83	Phlebitis in the lower limbs, hypertension, persecutory delusions, mild hypercholesterolemia, bilateral cataract.	Basic autonomy maintained only for feeding and walking. She begins with delusional thoughts, confusion and disorientation.
P5	84	Bilateral low vision, hypertension, hypothyroidism.	Impaired language deficit, level of collaboration not adequate to the context. Disoriented.
P6	69	Hyperphagia, chronic cerebral vasculopathy, hepatic steatosis.	Total aphasia, muscle stiffness. Constant walking.
P7	89	Low vision (right eye), prostatic hypertrophy.	Behavioural disorders characterized by constant complaining and fake crying. Slow walking.
P8	84	Hypercholesterolemia, sclero-hypertensive valvular heart disease. Wearing a pacemaker. Assumption of anticoagulants.	Walking with assistance. Behavioural disturbances and agitation. Basic autonomy totally compromised.
P9	82	Hypertension	Alternate moments of psychomotor agitation with total apathy and non-participation in activities.
P10	92	Arterial hypertension, hypertensive sclerotic heart disease, deforming spondylarthrosis, bilateral gonarthrosis, osteoporosis, chronic steatosis liver disease, chronic gastroduodenitis, irritable bowel and diverticulosis of the colon, atrial fibrillation in TA.	Apathetic. Slowed and assisted walking.
P11	72	Arterial hypertension, tendon retractions for loss of consciousness since the 1980s	Fluent speech but not adequate in content. Mixture of words and thoughts, constructive and ideomotor apraxia, impaired executive functions. Ideational replacement of familiar faces, psychomotor agitation, hyperphagia.
P12	78	Chronic vascular encephalopathy, thyroidectomy for goiter.	All cognitive domains are totally compromised. Fluent speech but not adequate in form and content.
P13	72	Cognitive decline with behavioural disorders.	Spatial disorientation, presence of persecutory delusions and hallucinations. Uncertain walking. Difficulty in understanding, impaired executive functions, attention deficit.

Participants	Age	Related pathology	Diagnosis
P14	75	Revascularized ischemic heart disease, Parkinson disease.	Wandering.
P15	85	Hypertensive ischemic heart disease in the compensation phase, eft femur arthroplasty, anxious syndrome, hearing loss.	Spatial disorientation, non-fluent speech. Good level of collaboration always adequate even if with opposite traits. Alternatively, inclined to the practice of motor exercises.
P16	78	Right breast cancer, severe osteoporosis.	Collaborating, normal walking. Fluent speech
P17	88	Arterial hypertension, hypercholesterolemia, osteoporosis, TIA carotid atheroma (left hemiparesis and dysarthria), hypoacusis.	Behavioural disturbances, insomnia, cooperation, fluent speech.
P18	79	Coronary artery bypass grafting, sleeplessness at night, mood changes and hallucinatory episodes.	Apathetic. Lack of socialization skills, constructive apraxia, non-fluent speech.
P19	82	Hypercholesterolemia, assumption of oral anticoagulants, bilateral hypoacusis, hyperuricemia, heart disease, hypertension and ischemic subject, cataract.	ADL (activities of daily living) kept at the limits of sufficiency. Fair ability to follow calculations and sentence completion.
P20	88	Sclero-degenerative heart disease, osteoporosis, hypovitaminosis B12, anaemia.	Unstable walking, knees vare, tendency to fall. Space-time disorientation. Collaborating but the level of socialization is not adequate.
P21	87	Ischemic heart disease, carotid atheromasia, benign prostatic hypertrophy, irritability and behavioural disturbances. From 3 years in a wheelchair.	Apathetic, uncooperative, he often closes his eyes and makes circular movements with his head<br>ADL (activities of daily living) kept for power only.
P22	71	Hypoacusis, insomnia, behavioural disorders, post head trauma aggression.	Adequate collaboration, fluent speech, daily activities maintained. Often fixed and absent gaze. Lack of socialization skills.
23	80	Hysterectomy and cholecystectomy, lumbar stenosis with multiple disc disease, depressive disorder.	Presence of behavioural disorders. Wandering, temporal disorientation, thought disturbances, apraxia, bradykinesia.
24	90	Memory disorders.	Collaborative, active, sociable. Deficit in space-time orientation and long-term memory.
25	79	Memory deficit, verbal aggression, irritability, disorder of thinking and behaviour. The psychotic symptoms are linked to personality disorder.	Collaborating, the behavioural symptomatology is kept under control by pharmacological and educational therapy. Autonomous walking. Disorientation. Good executive functions but presents ideomotor and constructive apraxia.
26	84	Bilateral hearing loss, hypertensive heart disease, diabetes mellitus (type 2).	Temporal disorientation, slow walking and with external support. Moderate cognitive impairment.
